# Pretreatment Neutrophil-to-Lymphocyte Ratio: A Prognostic Biomarker of Survival in Patients With Epithelial Ovarian Cancer

**DOI:** 10.7759/cureus.16429

**Published:** 2021-07-16

**Authors:** Sarah O John-Olabode, Kehinde S Okunade, Gbenga Olorunfemi, Adaiah Soibi-Harry, Garba Rimi, Benedetto Osunwusi, Adeyemi Okunowo, Lemchukwu Amaeshi, Rose Anorlu

**Affiliations:** 1 Haematology and Blood Transfusion, College of Medicine University of Lagos, Lagos, NGA; 2 Obstetrics and Gynaecology, Lagos University Teaching Hospital, Lagos, NGA; 3 Obstetrics and Gynaecology, College of Medicine University of Lagos, Lagos, NGA; 4 Epidemiology and Biostatistics, School of Public Health, University of Witwatersrand, Johannesburg, ZAF; 5 Internal Medicine, Lagos University Teaching Hospital, Lagos, NGA

**Keywords:** neutrophil-to-lymphocyte ratio, epithelial ovarian cancer, overall survival, progression - free survival, systemic inflammatory response syndrome

## Abstract

Background

Inflammation is pathognomonic of all stages of tumor formation, and therefore, there is renewed interest in systemic inflammatory response (SIR) markers including haematological inflammatory markers such as neutrophil-to-lymphocyte ratio (NLR) as prognostic predictors in several cancers.

Aim

This study was aimed to investigate the effect of pretreatment peripheral blood NLR on the survival prognosis of patients with epithelial ovarian cancer (EOC).

Methods

We identified 93 patients with a complete clinical record from a cohort of 155 patients who received treatment for EOC between 2009 and 2018. Patients’ sociodemographic and clinicopathologic characteristics, and updated three-year follow-up status were extracted from medical records. Pretreatment peripheral blood NLR was calculated by dividing the neutrophil count by the lymphocyte count. We employed the receiver operating characteristic (ROC) curve to identify the optimal cut-off value of the NLR in estimating progression-free survival (PFS) and overall survival (OS). The PFS and OS were assessed using the Kaplan-Meier method, and survival differences were compared using the Log Rank (Mantel-Cox) test. Independent prognostic predictors were determined using Cox regression analysis.

Results

According to the ROC curves, the optimal cut-off values for the NLR were 2.23 and 1.93 for PFS and OS, respectively. A high NLR was associated with poor PFS (P = 0.033) and OS (P = 0.013) in the univariate analyses. In the multivariate analyses, a high NLR was still an independent predictor of OS (hazard ratio [HR] = 2.23; 95% CI, 1.08 to 4.61) but not PFS (hazard ratio [HR] = 2.43; 95% CI, 0.95 to 6.27).

Conclusion

The NLR at an optimum cut-off value of 1.93 is an independent prognostic predictor of OS in patients with EOC.

## Introduction

About 90% of all histological types of ovarian cancer are of epithelial origin [1,2] with over 70% of epithelial ovarian cancer (EOC) being diagnosed at the advanced stage of the disease [3]. Despite the great progress in surgical intervention and chemotherapy, the prognosis of EOC remains poor due to the absence of specific symptoms in the early stage and the tendency of the disease to metastasize over a short period.

The interaction between inflammation and cancer development has gained significant research interest in the recent past [4]. Various hematological markers of inflammation could be useful in the prediction of survival in patients with different types of cancer [5-8]. Inflammation is seen in all stages of tumor formation including the initiation, promotion, progression, invasion, and metastasis of a tumor [9,10], and there is currently a renewed interest in systemic inflammatory response (SIR) markers including hematological inflammatory markers. One of such hematological inflammatory markers that have shown the potential to be a credible prognostic marker in various cancers including EOC is the neutrophil-to-lymphocyte ratio (NLR) [11-14].

NLR reflects the balance of the inflammatory and immune systems, and also indicates the balance between pro-tumor and anti-tumor status, making it a useful predictor of cancer prognosis [15]. NLR is a useful marker for evaluating the systemic balance between the pro-tumor inflammatory effect of neutrophil and the anti-tumor immune response of lymphocytes [16,17]. The use of biomarkers like NLR, which are inexpensive and readily available, as reliable prognostic markers are necessary for the identification of high-risk patients who may benefit from maintenance therapy following their upfront primary treatment with primary debulking surgery and adjuvant chemotherapy or neoadjuvant chemotherapy and interval debulking surgery. To our knowledge, there have been no studies to date among black African women on the prognostic significance of NLR in EOC. Hence, our study was aimed to evaluate the role of pretreatment peripheral blood NLR on the three-year prognostic outcomes of EOC patients managed in a public tertiary center in Lagos, Nigeria over a 10-year review period.

## Materials and methods

Study design and clinical setting

We designed a retrospective observational study involving a review of the ward register and medical records of women diagnosed with epithelial ovarian cancer (EOC) and who had their complete primary treatment at the Lagos University Teaching Hospital (LUTH), Lagos, Nigeria between March 2009, and February 2018. LUTH is the teaching hospital of the College of Medicine, University of Lagos. It acts as a tertiary referral center for other hospitals in Lagos and its surrounding states.

Eligibility criteria

**Figure 1:**
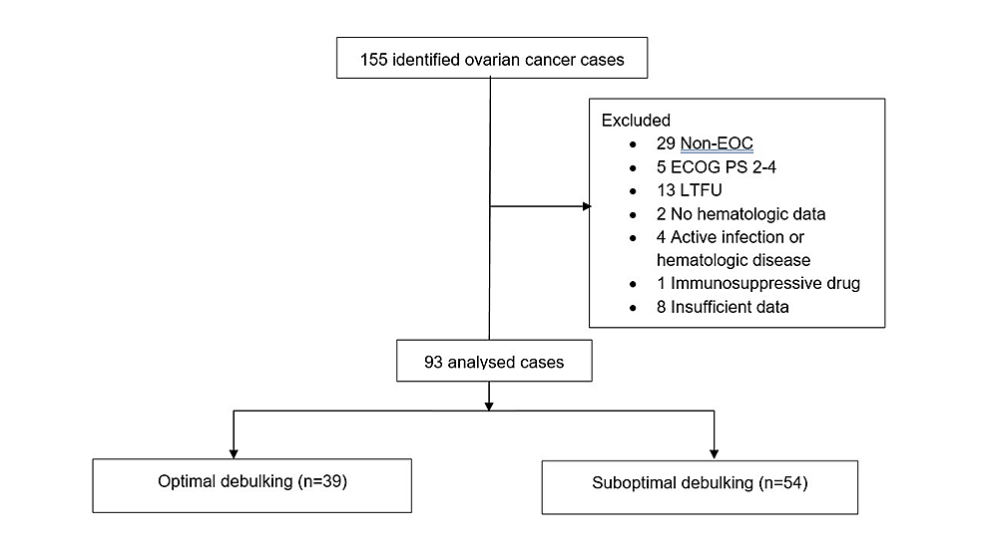
Flowchart of patient selection process. EOC: epithelial ovarian cancer; ECOG: Eastern Cooperative Oncology Group; PS: performance status; LTFU: lost to follow-up.

Ninety-three patients with complete clinical records were included in the analysis (Fig 1) Exclusion criteria were: (1) non-EOC; (2) Eastern Cooperative Oncology Group (ECOG) performance status of 2-4 [18]; (3) failure of completion of primary treatment or loss to follow-up; (4) unavailable pretreatment hematologic parameters; (5) record of disease complications such as an active infection; (6) any hematologic disease; or (7) medication with any immunosuppressive agent. Relevant data extracted from the ward register and patients’ clinical records were age, parity, body mass index (BMI), menopausal status, serum cancer antigen-125 (CA-125) concentration, comorbidities such as diabetes mellitus, hypertension, cardiac, liver, and kidney disease), complete blood count, presence of ascites, International Federation of Gynecology and Obstetrics (FIGO) stage, type of upfront treatment, surgical debulking status [19], histological subtypes [20], progression-free survival, and overall survival. We selected the pretreatment peripheral neutrophil and lymphocyte counts from the complete blood counts. The NLR was then estimated by dividing the absolute neutrophil count by the lymphocyte count [21].

Study outcomes

Two outcomes were assessed: (1) progression-free survival (PFS), determined by estimating the time interval from the completion of primary treatment to the first relapse as evidenced by clinical examination, elevated tumor marker (serum cancer antigen-25 levels) and/or radiological studies; and (2) overall survival (OS), defined by determining the time interval from the completion of primary treatment until the death of the patients from all causes or last follow-up for patients who were still alive [22]. We censored the survival data after three-year follow-up.

Sample size determination

We used G*Power for Windows version 3.1.9.2 (Kiel University, Germany) to calculate the sample size. The study was powered for a two-sided test with a Type I error rate of 5% (Zα=1.96) and power of 80% (Zβ=0.84), adjusted for a 10% attrition rate. Given a variability of 0.5 in the hazard ratio (HR) of death and progression of the disease from a high NLR of 1.91 and 1.82, respectively [23], the number of deaths was 75 and while that of disease relapse was 88. However, being a retrospective study, records of all eligible EOC patients during the study period were reviewed and included for data analyses.

Statistical analysis

Data were analyzed using SPSS version 27.0 statistical package for Windows (manufactured by IBM Corp., Armonk, NY, United States) and descriptive statistics were computed for all patients’ baseline characteristics. Patients’ sociodemographic and clinicopathologic characteristics were described if normally distributed using mean and standard deviation (SD) and if skewed using median and interquartile range (IQR), for continuous variables. Categorical variables were presented using frequencies and percentages. The optimum cut-off values for the NLR were estimated using the Receiver operating characteristic (ROC) curve analysis with Youden's index. Estimates of PFS and OS time stratified by NLR ratios were determined using the Kaplan-Meier (KP) curve analysis and then compared by the use of Log Rank (Mantel-Cox) test statistic [24]. We censored patients without tumor recurrence or those who were alive at the last follow-up. Hazard ratios (HR) were determined using the multivariate Cox regression models while adjusting for other potential covariates. We built the final multivariate models to include the patients’ age and other variables using a P-value < 0.5 to remain in the model. Statistical significance was reported at P-value < 0.05. 

Ethical considerations

This study was conducted with consideration for the provisions of the Declaration of Helsinki. The Health Research Ethics committee of the Lagos University Teaching Hospital approved the protocol (ADM/DCST/HREC/APP/3699) before access to the patients’ case records for data collection and analysis.

## Results

Out of the 155 patients with ovarian cancer who were managed in the hospital during the period under review, we included 93 patients’ data in the final analyses. We excluded 29 women with non-EOC, 5 with an ECOG performance status of between 2 and 4, 13 who were lost to follow-up or failed to complete their primary treatment, 2 with unavailable pretreatment hematologic parameters, 4 with an active infection or hematologic disease, 1 on medication with an immunosuppressive agent and 8 with insufficient clinical data (Figure 1).

The mean age of the patients at presentation was 47.1 years (SD: 13.9 years). A total of 57 (63.1%) women had primary surgical debulking as their upfront primary treatment with the majority diagnosed with advanced FIGO stages of the disease (n = 65, 69.8%), and high-grade serous carcinomas (n = 54, 58.1%). Twenty-nine (31.2%) of the patients had documented tumor relapse while 36 (38.7%) died during the follow-up period (Table 1).

**Table 1:** Characteristics of patients with epithelial ovarian cancer (n = 93). HGSC: high-grade serous carcinomas; IQR: interquartile range; NLR: neutrophil-to-lymphocyte ratio; LGSC: low-grade serous carcinomas; NACT: neoadjuvant chemotherapy; PDS: primary debulking surgery; SD: standard deviation.

Characteristics	Number (%)
Mean age (SD) in years	47.1 (13.9)
Mean BMI (SD) in kg/m^2 ^	23.6 (5.2)
Median serum CA-125 levels (IQR) in U/mL	112.4 (44.2, 582.1)
Median NLR (IQR)	1.89 (1.21, 3.12)
Parity	
Nulliparous	43 (46.2)
Multiparous	50 (53.8)
Menopausal status	
Premenopause	52 (55.9)
Postmenopause	41 (44.1)
Comorbidity	
Yes	16 (17.2)
No	77 (82.8)
Upfront primary treatment	
PDS	57 (61.3)
NACT	36 (38.7)
Ascites	
Yes	37 (39.8)
No	56 (60.2)
FIGO stage	
Early (I & II)	28 (30.2)
Advanced (III & IV)	65 (69.8)
Surgical debulking status	
Optimal	39 (41.9)
Suboptimal	54 (58.1)
Histological subtype	
Type I (LGSC and others)	33 (35.5)
Type II (HGSC)	60 (64.5)

**Figure 2:**
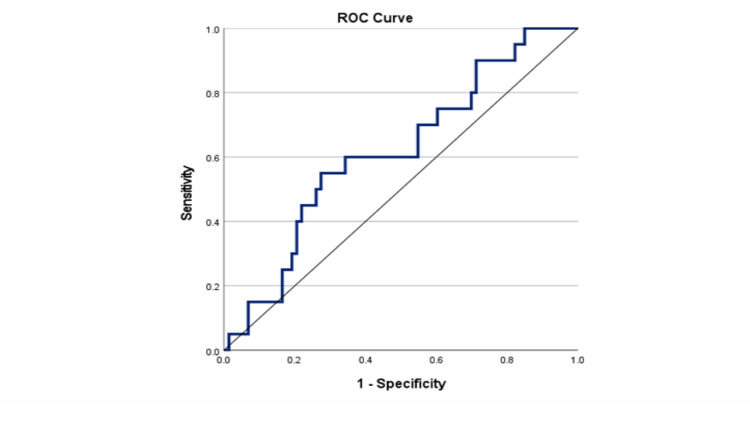
Receiver operating curve of pre-operative NLR for predicting PFS in patients with EOC. ROC: receiver operating curve; NLR: neutrophil-to-lymphocyte ratio; PFS: progression-free survival; EOC: epithelial ovarian cancer.

**Figure 3:**
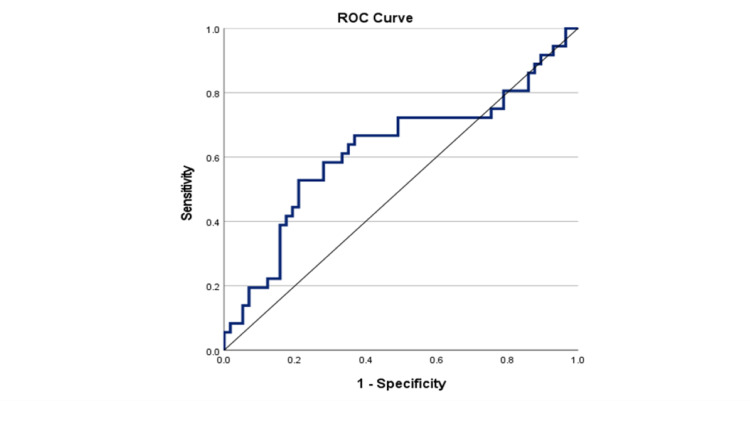
Receiver operating curve of pre-operative NLR for predicting OS in patients with EOC. ROC: receiver operating curve; NLR: neutrophil-to-lymphocyte ratio; OS: overall survival; EOC: epithelial ovarian cancer.

As shown in Figure 2, the area under the curve (AUC) was 0.68 (95% CI: 0.57-0.80; P = 0.005), and the optimal NLR cut-off value for PFS was 2.23, with the sensitivity and specificity being 0.66 and 0.72, respectively. There were 55 (59.1%) patients with an NLR < 2.23 and 68 (44.2%) patients with an NLR ≥ 2.23. In Figure 3, we recorded the AUC as 0.62 (95% CI: 0.50-0.75; P = 0.049), and the optimal NLR cut-off value for OS was 1.93, with the sensitivity and specificity being 0.67 and 0.63, respectively. Using this cut-off value, there were 48 (51.6%) patients with an NLR<1.93 and 45 patients (48.4%) with an NLR≥1.93.

**Figure 4:**
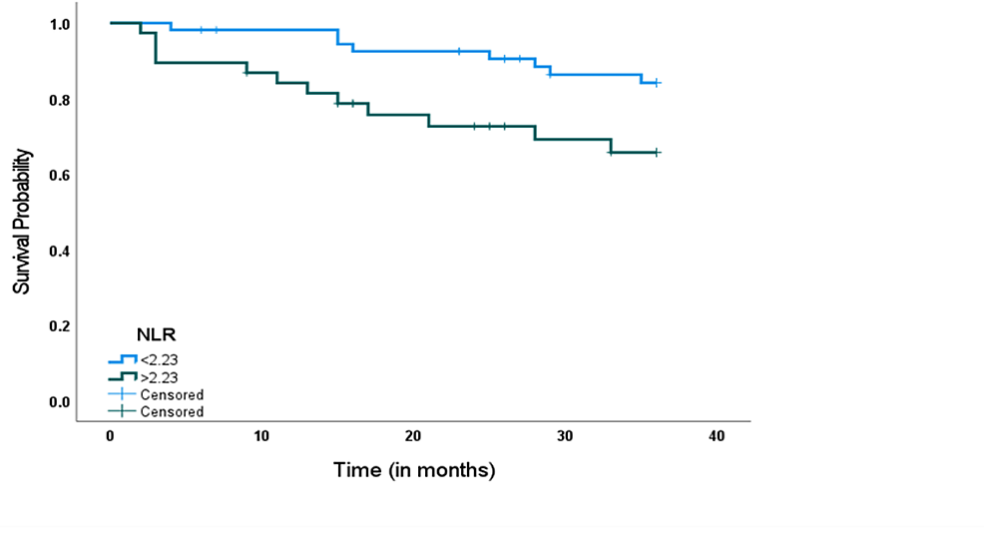
Kaplan-Meier survival curves for PFS in patients with EOC after surgical resection. Progression-free survival of patients with NLR > 2.23 was shorter than those with NLR ≤ 2.23 (P = 0.033). NLR: neutrophil-to-lymphocyte ratio; PFS: progression-free survival; EOC: epithelial ovarian cancer.

**Figure 5:**
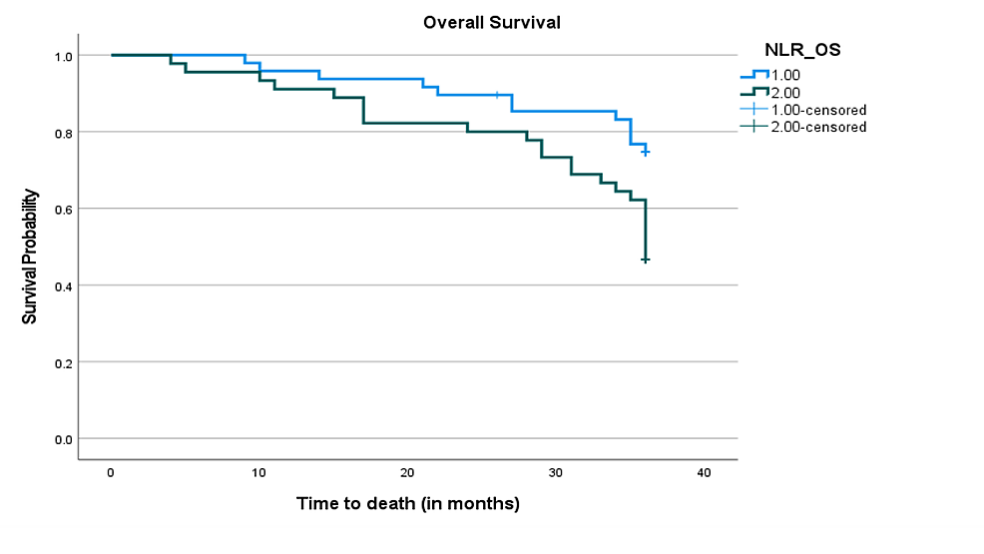
Kaplan-Meier survival curves for OS in patients with EOC after surgical resection. Progression-free survival of patients with NLR > 1.93 was shorter than those with NLR ≤ 1.93 (P = 0.013). NLR: neutrophil-to-lymphocyte ratio; OS: overall survival; EOC: epithelial ovarian cancer.

In the Kaplan-Meier (KP) survival curve stratified by the NLR, we recorded statistically shorter PFS and OS in EOC patients with NLR values above the optimal cut-offs than in those with an NLR below the cut-offs (Figures 4, 5).

In the univariate Cox regression analysis, parity (P = 0.013) and pretreatment NLR (P = 0.033) were significant prognostic factors for PFS, whereas, in the multivariate analysis, only parity (HR: 4.63, 95% CI: 1.39-15.39, P = 0.012) was an independent predictor of reduced PFS (Table 2). In Table 3, parity (P = 0.031) and pretreatment NLR (P = 0.013) were recorded to be significant prognostic predictors of OS in the univariate analyses, and following adjustment in the final multivariate model both parity (HR: 2.32, 95% CI: 1.11-4.84, P = 0.025) and NLR (HR: 2.23, 95% CI: 1.08-4.61, P = 0.031) were also recorded to be independent risk predictors of reduced OS.

**Table 2:** Univariate and multivariate analyses for progression-free survival. BMI, body mass index; HR, hazard ratio; NACT; neoadjuvant chemotherapy; NLR, neutrophil-to-lymphocyte ratio; PDS; primary debulking surgery; FIGO: International Federation of Gynecology and Obstetrics; CA-125: cancer antigen-125; LG includes endometrioid carcinoma, clear cell carcinoma, mucinous carcinoma, and low-grade serous carcinomas; HG includes high-grade serous carcinomas.

Characteristics	Category	Univariate	Multivariate	
		P-value	HR (95% CI)	P-value
Age	≥47 vs. <47 years	0.894	0.60 (0.22-1.63)	0.316
Parity	Multiparous vs. nulliparous	0.013	4.63 (1.39-15.39)	0.012
Menopausal status	Postmenopause vs. premenopause	0.632	-	-
BMI	≥24.0 vs. <24.0 kg/m^2^	0.990	-	-
Serum CA-125 levels	≥112.0 vs. <112.0 U/mL	0.401	1.30 (0.36-4.68)	0.687
Comorbidity	Yes vs. No	0.069	2.77 (0.91-8.40)	0.072
Upfront primary treatment	NACT vs. PDS	0.366	0.71 (0.23-2.15)	0.539
Ascites	Yes vs. No	0.294	0.91 (0.34-2.40)	0.848
FIGO stage	Advanced vs. early	0.296	1.19 (0.25-5.74)	0.824
Surgical debulking status	Optimal vs. suboptimal	0.952	-	-
Histological subtype	HG vs. LG	0.958	-	-
Pretreatment NLR	≥2.23 vs. <2.23 years	0.033	2.43 (0.95-6.27)	0.065

**Table 3:** Univariate and multivariate analyses for overall survival. BMI, body mass index; HR, hazard ratio; NACT; neoadjuvant chemotherapy; NLR, neutrophil-to-lymphocyte ratio; PDS; primary debulking surgery; FIGO: International Federation of Gynecology and Obstetrics; CA-125: cancer antigen-125; LG includes endometrioid carcinoma, clear cell carcinoma, mucinous carcinoma, and low-grade serous carcinomas; HG includes high-grade serous carcinomas.

Characteristics	Category	Univariate	Multivariate	
		P-value	HR (95% CI)	P-value
Age	≥47 vs. <47 years	0.712	0.65 (0.32-1.32)	0.230
Parity	Multiparous vs. nulliparous	0.031	2.32 (1.11-4.84)	0.025
Menopausal status	Postmenopause vs. premenopause	0.927	-	-
BMI	≥24.0 vs. <24.0 kg/m^2^	0.795	-	-
Serum CA-125 levels	≥112.0 vs. <112.0 U/mL	0.662	-	-
Comorbidity	Yes vs. no	0.749	-	-
Upfront primary treatment	NACT vs. PDS	0.657	-	-
Ascites	Yes vs. No	0.284	1.05 (0.53-2.07)	0.900
FIGO stage	Advanced vs. early	0.507	-	-
Surgical debulking status	Optimal vs. suboptimal	0.944	-	-
Histological subtype	HG vs. LG	0.470	1.22 (0.57-2.60)	0.615
Pretreatment NLR	≥1.93 vs. <1.93 years	0.013	2.23 (1.08-4.61)	0.031

## Discussion

Currently, the estimate of the survival outcomes of patients with EOC relies mainly on various clinicopathological variables, such as the extent of tumor resectability during debulking surgery, FIGO stage, presence of ascites and serum cancer antigen 125 levels [22]. While reflecting the behavior and presentation of cancer in biology, these parameters may not necessarily represent the actual burden of the disease in patients with EOC. Recently, there is increased attention on NLR, which is easily obtained from the routine pretreatment peripheral blood tests, as an important prognostic marker in multiple cancers Including EOC [11-14]. Therefore, in the present study, we evaluated the impact of pretreatment peripheral blood NLR on the short-term survival outcomes of EOC patients managed over a 10-year review period at LUTH, and we found that a high pretreatment NLR was an independent predictor of reduced OS, whereas its prognostic effect on PFS was only dependent on the patient’s parity.

The NLR is an inflammation- and immunity-related marker calculated using the ratio of peripheral absolute neutrophil count to lymphocyte count. This reflects both the neutrophilia and lymphopenia within the tumor microenvironment. The increase in neutrophils supports cancer cell invasion, migration, and angiogenesis which lead to cancer progression [25] while the reduction in lymphocytes results from a poor and inadequate immunologic response to the tumor [26]. In corroboration with the findings of other published studies [27,28] suggesting that the NLR is an independent prognostic predictor of survival, our study found that the different survival endpoints have different cut-off values and that the NLR has a significant prognostic value in EOC patients. The lack of significant independent prognostic effect of NLR on the OS may likely due to the relatively short follow-up period of three years used in our current study compared to the average five-year follow-up used in other similar studies [27-29]. This may also be due to the optimum cutoff value selected in the current study to stratify NLR into the high-NLR and low-NLR groups. In previous studies, the cutoff values of NLR were estimated using methods such as the median levels or ROC analysis for PFS and OS [27,28]. Thus, it is still difficult to determine which of these is the best method. The NLR cutoff value in our study was selected from the ROC analysis, and this was used to stratify the EOC patients into low- and high-NLR groups. However, our finding was also in line with that of Raungkaewmanee et al. [29] who reported no significant association between NLR and PFS or OS in ovarian cancer patients. Williams et al. [30] reported in their study conducted that higher NLR levels were associated with various clinicopathological characteristics including the presence of ascites, tumor stage and grade; however, similar associations were not seen in our study. 

The major limitations of this study were the poor clinical data management system in our center resulting in the high number of ovarian cancer cases with incomplete or missing data. Furthermore, as this is an institutional-based study, we may not be able to generalize the findings to other centers or geopolitical zones in the country. Therefore, there is a need to further evaluate the clinical applicability and identify gaps in this study through a long-term prospective, multicenter study with a larger sample size.

## Conclusions

Our study reported that pretreatment peripheral blood neutrophil-lymphocyte ratio at optimum cut-off is an important prognostic predictor of PFS in epithelial ovarian cancer. However, a prevalence study among healthy cancer-free women should be conducted to determine the normal range of NLR, as this would help to define the optimum cut-off for NLR levels in subsequent studies. In addition, there is a need to provide additional evidence to corroborate the findings of this study through a prospective multicenter study with a larger sample size among black women in sub-Saharan Africa.
